# Ultrahigh-resolution 7-Tesla anatomic magnetic resonance imaging and diffusion tensor imaging of *ex vivo* formalin-fixed human brainstem-cerebellum complex

**DOI:** 10.3389/fnhum.2024.1484431

**Published:** 2024-11-27

**Authors:** Sahin Hanalioglu, Siyar Bahadir, Ahmet C. Ozak, Kivanc Yangi, Giancarlo Mignucci-Jiménez, Muhammet Enes Gurses, Alberto Fuentes, Ethan Mathew, Dakota T. Graham, Muhammed Yakup Altug, Egemen Gok, Gregory H. Turner, Michael T. Lawton, Mark C. Preul

**Affiliations:** ^1^The Loyal and Edith Davis Neurosurgical Research Laboratory, Department of Neurosurgery, Barrow Neurological Institute, St. Joseph's Hospital and Medical Center, Phoenix, AZ, United States; ^2^Department of Neurosurgery, Hacettepe University, Ankara, Türkiye; ^3^Department of Neurosurgery, Akdeniz University, Antalya, Türkiye; ^4^Department of Neurosurgery, Turkish Republic Ministry of Health, University of Health Sciences, Prof. Dr. Cemil Tascioglu City Hospital, Istanbul, Türkiye; ^5^Neuroimaging Innovation Center, St. Joseph's Hospital and Medical Center, Barrow Neurological Institute, Phoenix, AZ, United States; ^6^Thurston Innovation Center, St. Joseph's Hospital and Medical Center, Barrow Neurological Institute, Phoenix, AZ, United States; ^7^Center for In Vivo Imaging and Therapeutics, St. Jude Children's Research Hospital, Memphis, TN, United States

**Keywords:** brainstem, cerebellum, diffusion tractography, formalin-preserved brain, magnetic resonance imaging, neuroanatomy

## Abstract

**Introduction:**

Brain cross-sectional images, tractography, and segmentation are valuable resources for neuroanatomical education and research but are also crucial for neurosurgical planning that may improve outcomes in cerebellar and brainstem interventions. Although ultrahigh-resolution 7-Tesla (7T) magnetic resonance imaging (MRI) and diffusion tensor imaging (DTI) reveal such structural brain details in living or fresh unpreserved brain tissue, imaging standard formalin-preserved cadaveric brain specimens often used for neurosurgical anatomic studies has proven difficult. This study sought to develop a practical protocol to provide anatomic information and tractography results of an *ex vivo* human brainstem-cerebellum specimen.

**Materials and methods:**

A protocol was developed for specimen preparation and 7T MRI with image postprocessing on a combined brainstem-cerebellum specimen obtained from an 85-year-old male cadaver with a postmortem interval of 1 week that was stored in formalin for 6 months. Anatomic image series were acquired for detailed views and diffusion tractography to map neural pathways and segment major anatomic structures within the brainstem and cerebellum.

**Results:**

Complex white matter tracts were visualized with high-precision segmentation of crucial brainstem structures, delineating the brainstem-cerebellum and mesencephalic-dentate connectivity, including the Guillain-Mollaret triangle. Tractography and fractional anisotropy mapping revealed the complexities of white matter fiber pathways, including the superior, middle, and inferior cerebellar peduncles and visible decussating fibers. 3-dimensional (3D) reconstruction and quantitative and qualitative analyses verified the anatomical precision of the imaging relative to a standard brain space.

**Discussion:**

This novel imaging protocol successfully captured the intricate 3D architecture of the brainstem-cerebellum network. The protocol, unique in several respects (including tissue preservation and rehydration times, choice of solutions, preferred sequences, voxel sizes, and diffusion directions) aimed to balance high resolution and practical scan times. This approach provided detailed neuroanatomical imaging while avoiding impractically long scan times. The extended postmortem and fixation intervals did not compromise the diffusion imaging quality. Moreover, the combination of time efficiency and ultrahigh-resolution imaging results makes this protocol a strong candidate for optimal use in detailed neuroanatomical studies, particularly in presurgical trajectory planning.

## 1 Introduction

The human brain, an intricate web of neural connections, has long been the subject of intense scientific scrutiny. Better visualization of the brain's complex structure is of crucial importance for neuroscience studies and neurosurgical procedures and has become a trending topic in neuroanatomical surgical research in recent years (Hanalioglu et al., 2022; Gonzalez-Romo et al., 2023; Shepherd and Hoch, 2022; Gurses et al., 2023, 2022). Traditional methods to visualize the brain's complex architecture have offered detailed microscopic perspectives obtained from conventional histological techniques. However, broader and more sophisticated digital views can be obtained through magnetic resonance imaging (MRI). Each approach comes with a unique set of challenges. Classical histology delivers high-resolution images through laborious procedures that may risk tissue distortion, compromising the structural integrity of the specimen (Alkemade et al., 2023; Casamitjana et al., 2022; Maranzano et al., 2020). Despite the digital flexibility in postprocessing and advanced technological manipulations that MRI offers, its spatial resolution has historically been inferior to that of optical microscopy, limiting its ability to capture fine neuroanatomical details. Microscopy can image much smaller structures with higher precision, particularly at the cellular and subcellular level, whereas MRI often struggles to resolve the intricate details of neuroanatomy (Dinse et al., 2013; Fiel et al., 1991; Vasung et al., 2017).

Bridging the resolution gap between histology and MRI, “magnetic resonance histology” combines the strengths of MRI's digital nature with longer scan times, the use of exogenous contrast agents, and specialized imaging hardware (Johnson et al., 2002). This technique can produce images that reveal structural details inaccessible using conventional *in vivo* human scans. However, *ex vivo* imaging studies of the human brain and brainstem using this technique have often been limited to specific anatomical sub-regions or focused predominantly on white matter because the high-resolution capability of histology is primarily effective when used to examine small structures under magnification (Johnson et al., 2002; Cappellen van Walsum and Henssen, 2022; Henssen et al., 2019; Edlow et al., 2023; Schira et al., 2023).

We introduce a novel technique that combines the precision of magnetic resonance microscopy with the depth and scope of diffusion tractography. This combination provides comprehensive, high-resolution, 3-dimensional (3D) virtual imaging of the human brainstem, including specific white matter pathways. Our findings can serve as a tool for studying the human brainstem and its complex neuroanatomy. Detailed examination of the brainstem anatomy can also aid in a better understanding of the microsurgical anatomy of various neurosurgical approaches to the brainstem and cerebellum (Deshmukh et al., 2006; Aydin et al., 2018; Wu et al., 2010; Jittapiromsak et al., 2008). Future enhancements to our technique can play a pivotal role in neuroanatomical research and neurosurgical planning, including the advanced study of brainstem safe entry zones, cerebellar peduncles, brainstem-cerebellar connectivity, and other relevant networks (Guberinic et al., 2022; Inci and Baylarov, 2024; Serrato-Avila et al., 2022; Yang et al., 2019; Cavalcanti et al., 2016).

## 2 Materials and methods

### 2.1 Specimen preparation and initial processing

**Figure 1:**
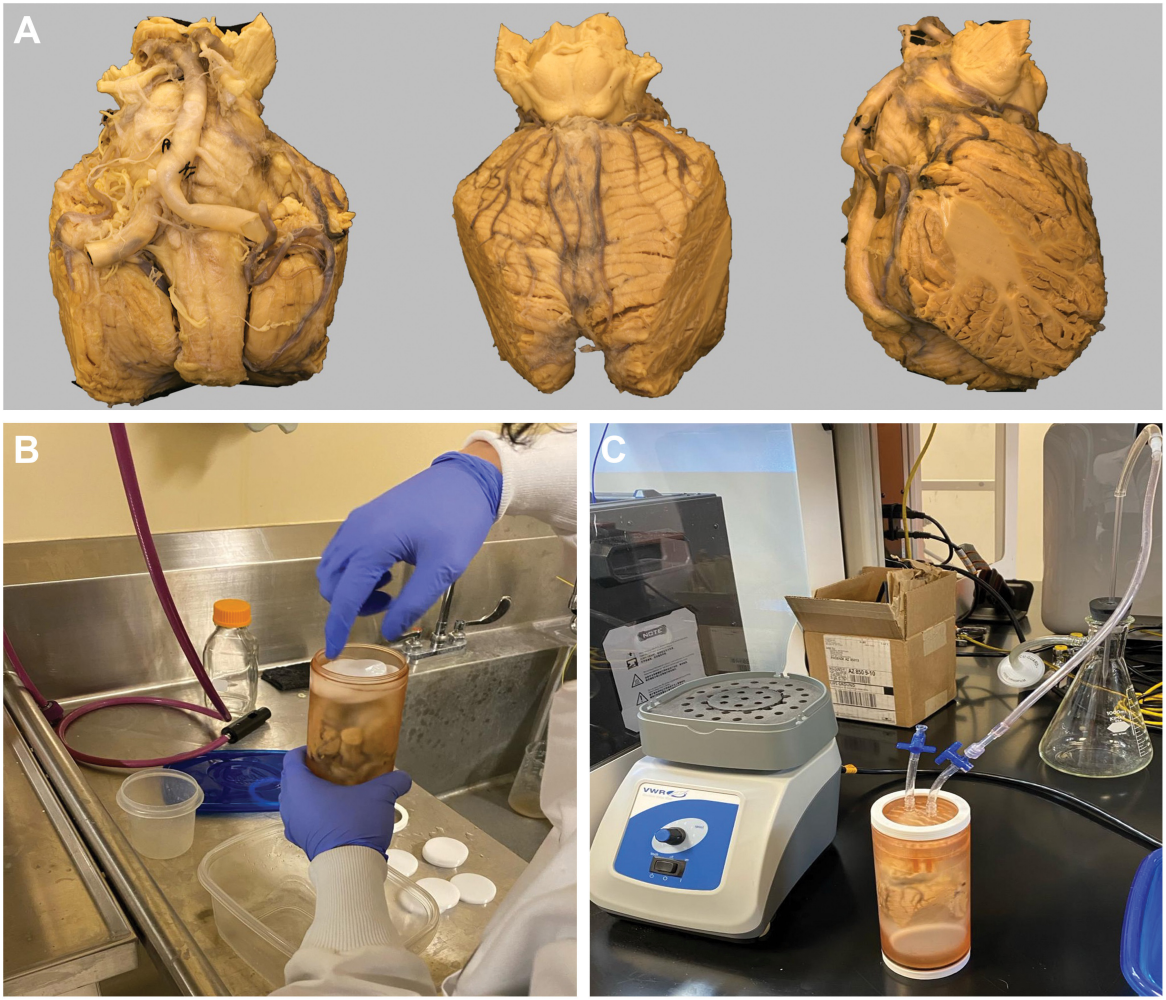
Cadaveric brainstem and cerebellum specimen, 3D-printed container, and the degassing procedure. **(A)** Anterior **(left)**, posterior **(middle)**, and lateral **(right)** views of the cadaveric brainstem and cerebellum gross specimen. **(B)** Placement of specimen in the custom-designed, 3D-printed container. The container was then filled with Fomblin Y-L-VAC 25/6 PFPE Pump Inert Oil (Subspecialty Fluids Co., Castaic, CA) and nonradiosensitive oval white spacers to reduce the volume of the inert solution used. **(C)** The degassing process shows the specimen in the container with dual outlet ports connected to a 3-way stopcock. The container was sealed with white oil-resistant Buna-N Rubber O-rings to ensure an airtight closing. *Used with permission from Barrow Neurological Institute, Phoenix, Arizona*.

A brainstem-cerebellum section was acquired from the brain of an 85-year-old male cadaver with a postmortem interval (PMI) of 174 h (7 days) (Figure 1A). The whole-brain specimen was preserved in formalin for 6 months before imaging and then rehydrated for 1 week in a standard saline solution to maintain structural integrity. Ethical review and approval were not required for the study on human participants in accordance with the local legislation and institutional requirements. Written informed consent from the participant's next of kin was not required in accordance with the national legislation and the institutional requirements. The brain specimen was unidentifiable except for sex, age, medical history, and cause of death. There was no history of neurological disease or condition. The tissue specimen was acquired from a reputable nonprofit research tissue provider (Science Care, Phoenix, AZ) approved by and agreed to by our institution (St. Joseph's Hospital and Medical Center, Phoenix, AZ).

The lateral portions of the cerebellum were sharply cut, facilitating a secure fit within the custom-designed, 3D-printed container (outer diameter: 6.8 cm) (Figure 1B). This container was tailored to fit the MRI bore using a 12-cm gradient insert. The container featured dual outlet ports connected to a 3-way stopcock (Figure 1C). The container was filled with Fomblin Y-L-VAC 25/6 PFPE Pump Inert Oil (Subspecialty Fluids Co., Castaic, CA) and nonradiosensitive spacers (Figure 1B) to reduce the volume of the inert solution used.

### 2.2 Degassing and vibration

The degasser was designed with a central cylinder featuring external threads on both ends and was sealed with internal rubber O-rings (Figure 1C). This setup, including a concave surface on the plugs, directed air bubbles out of the container, with 1 plug incorporating 2 ports for fluid management during the degassing process, followed by continuous vibration using a standard vortex mixer (VWR Scientific Products, Radnor, PA). The cylinder and plugs were manufactured using a Formlabs Form 2 SLA 3D printer with BioMed Amber Resin. The caps were produced on a Flashforge Creator Pro FDM 3D printer using polylactic acid. Oil-resistant Buna-N Rubber O-rings were used to ensure an airtight seal (Figure 1C).

### 2.3 Image acquisition and processing

Diffusion tensor imaging (DTI) lasted 48 h and 48 min, and subsequent anatomical imaging was performed using a 7-Tesla (7T) MRI system (Bruker Biospec 70/30 with 30-cm bore size) equipped with a 70-mm volume coil. Initially, DTI images were obtained to map neural pathways using a high *b*-value of 3,105 s/mm^2^ to enhance contrast and resolution. The gradient settings with a duration (Δ) of 7 ms and a separation (δ) of 14 ms were selected to capture the microstructural features of the brainstem and cerebellum. The in-plane resolution was set at 0.55 mm with a slice thickness of 0.5 mm to obtain high-resolution imaging conducive to fiber analysis and tractography.

**Figure 2:**
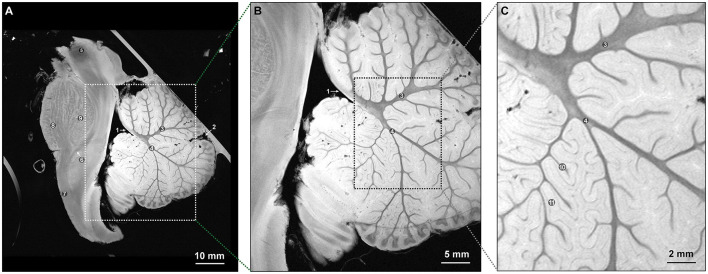
Midsagittal T2-weighted FLASH sequence MR images of the specimen at ultrahigh resolutions allowing for detailed visualization (TR/TE: 3500/15 ms). **(A)** Visualization of crucial anatomical structures and general architecture of the brainstem-cerebellum complex, 10-mm anatomic section. **(B)** Visualization of the arbor vitae and general architecture of the cerebellum, 5-mm section. **(C)** Visualization of the intralobular architecture of the cerebellum, 2-mm section. 1: Roof of the fourth ventricle. 2: Primary fissure of the cerebellum. 3: Apical arm of the arbor vitae. 4: Central arm of the arbor vitae. 5: Red nucleus. 6: Medial lemniscus. 7: Pyramidal tract. 8: Longitudinal fasciculus pons. 9: Transverse pontine fibers. 10: Secondary sulci. 11: Secondary folds of the cerebellum. *Used with permission from Barrow Neurological Institute, Phoenix, Arizona*.

**Figure 3:**
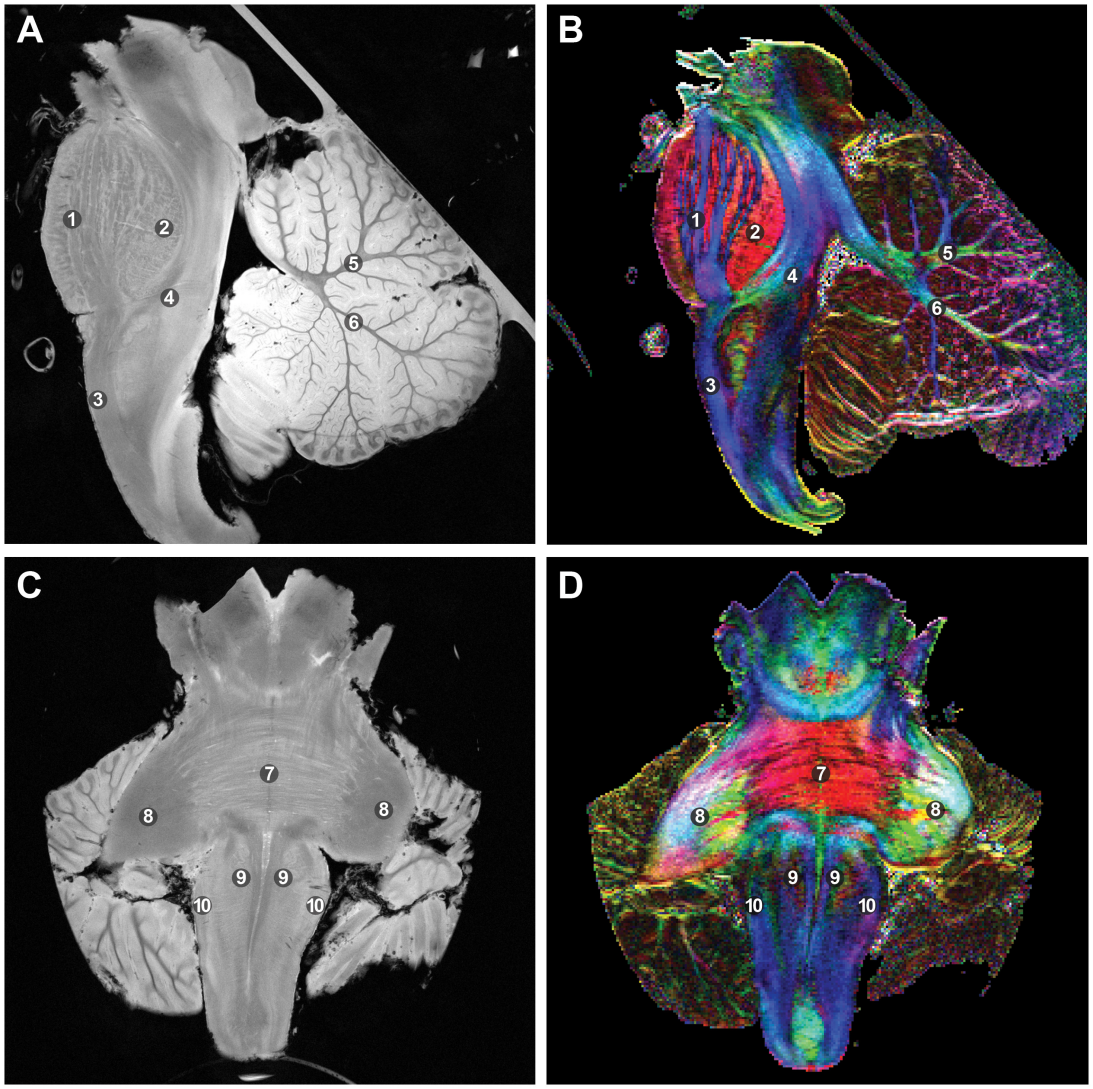
T2-weighted fast low-angle shot sequence MR images with an in-plane resolution of 0.55 mm and fractional anisotropy (FA) color mapping, which delineates and separates crucial structures. The coloration in the FA map corresponds to local tract direction: red denotes left-right, blue signifies inferior-superior, and green indicates anterior-posterior. **(A)** Mid-sagittal view. **(B)** Corresponding fractional anisotropy color mapping. **(C)** Mid-coronal view. **(D)** Corresponding color mapping. 1: Longitudinal fasciculus pons. 2: Transverse fasciculus pons. 3: Pyramidal tract. 4: Medical lemniscus. 5: Apical arm of the cerebellar arbor vitae. 6: Central arm of the cerebellar arbor vitae. 7: Pontocerebellar fibers. 8: Brachium pontis. 9: Pyramids. 10: Olive. *Used with permission from Barrow Neurological Institute, Phoenix, Arizona*.

After DTI, high-resolution anatomical images were acquired using a fast low-angle shot sequence (Figures 2, 3), with TR/TE set to 1,500/9 ms for axial and coronal views and 3,500/15 ms for sagittal views. This step, performed after diffusion imaging, provided precise anatomical references and facilitated the accurate overlay of diffusion data onto the structural images.

### 2.4 Tractography

A deterministic algorithm within DSI Studio (https://dsi-studio.labsolver.org/) was used to identify and track neural fibers (Yeh et al., 2010; Abhinav et al., 2014; Wade et al., 2021; Yeh et al., 2013; Yeh and Tseng, 2013; Yeh et al., 2011). DTI data were used to inform the placement of more than 1 million seeding points, ensuring detailed visualization of the brain's connectivity. Fibers not meeting the specified length requirements were removed by filtration.

### 2.5 Postimaging processing

Imaging data acquired in multiple contiguous blocks were combined by aligning the 3D volumes from each scan in their correct order. This alignment process effectively mirrored a continuous acquisition process and constructed an accurate 3D model of the brainstem-cerebellum complex.

DIPY (https://dipy.org/) was used to generate diffusion and fractional anisotropy (FA) maps to quantify the directional preference of water diffusion within tissues. FA is calculated from the diffusion tensor, indicating the extent of preferential water diffusion along the principal direction vs. perpendicular axes. These maps were used to validate the anatomical accuracy of our models against established Montreal Neurological Institute (MNI) space standards in terms of spatial orientation and resolution (Aggarwal et al., 2013).

### 2.6 Anatomic segmentation

Segmentation was performed on the basis of high-resolution images, references from histological images in *Gray's Anatomy* (Gray et al., 2005), FA, and diffusion-weighted imaging of the specimen at relevant locations. Clinical and anatomical significance was considered when selecting the segmented structures, and references from Rhoton's *Cranial Anatomy and Surgical Approaches* (Rhoton, 2003) and *Gray's Anatomy* (Gray et al., 2005) were consulted for better delineation of anatomical and topological extensions (Table 1) (Gray et al., 2005; Rhoton, 2003).

**Table 1:** The 31 segmented regions of interest and their corresponding anatomical structures.

**Region**	**Segmented regions of interest**
Mesencephalon	Red nucleus
	Substantia nigra
	Superior colliculus
	Inferior colliculus
	Trochlear nucleus
	Oculomotor nucleus
	Mesencephalic nucleus of trigeminal
	Cerebral crus
	Periaqueductal gray
	Superior cerebellar peduncle
Pons	Abducens nucleus
	Facial nucleus
	Motor nucleus of trigeminal
	Principal nucleus of trigeminal
	Vestibular complex^*^
	Middle cerebellar peduncle
Medulla	Vestibular complex^*^
	Spinal nucleus of the trigeminal nerve
	Nucleus tractus solitarius
	Nucleus gracilis
	Nucleus cuneatus
	Inferior olivary complex
	Hypoglossal nucleus
	Dorsal vagal nucleus
	Accessory nucleus
	Inferior cerebellar peduncle
Cerebellum	Cerebellar cortex
	Vermis
	Dentate nucleus
White matter	Medial lemniscus
	Medial longitudinal fasciculus
	Corticospinal tract

^*^The vestibular complex is included in both the pons and the medulla.

3D Slicer (https://www.slicer.org/) was used for segmentation and reconstruction. Segmentation was primarily oriented in the transverse plane, which is closer to the appearance of histological images. Orientation in the sagittal and coronal planes, along with the “smooth” module within 3D Slicer, were used where necessary to reduce slice-to-slice inconsistency. The color codes for the segmented anatomical regions are given in Supplementary Table 1.

## 3 Results

### 3.1 Qualitative results: tractography and anatomical features

#### 3.1.1 Mapping and streamline generation

**Figure 4:**
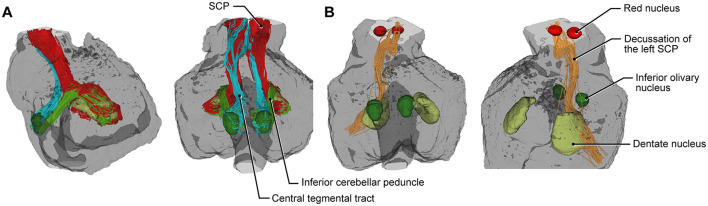
Tractography results delineating the mesencephalic-dentate connectivity. **(A)** Tractography of the specimen demonstrates the left superior cerebellar peduncle (SCP, *red*), inferior cerebellar peduncle (*light green*), and central tegmental tract (*light blue*). **(B)** Right-sided images showing the nuclei and tracts involved in the Guillain-Mollaret triangle, decussation of the left SCP (*orange*), red nucleus (*red*), inferior olivary nuclei (*dark green*), and dentate nuclei (*light green*). *Used with permission from Barrow Neurological Institute, Phoenix, Arizona*.

FA mapping and tractography revealed the intricacies of crucial anatomical structures, including white matter fiber pathways extending from the mesencephalon to the medulla, crossing the pons, and forming the fasciculi of the superior cerebellar peduncle (SCP), middle cerebellar peduncle (MCP), and inferior cerebellar peduncle (ICP), as well as visible decussation fibers (Figures 3, 4). Tractography thus provided insights into mesencephalic-dentate connectivity, emphasizing the detailed pathways and their anatomical significance. This connectivity was specifically demonstrated and analyzed within the context of the Guillain-Mollaret triangle, focusing on the anatomical structures that form this triangle (Figure 4).

#### 3.1.2 Spatial registration and 3D reconstruction

**Figure 5:**
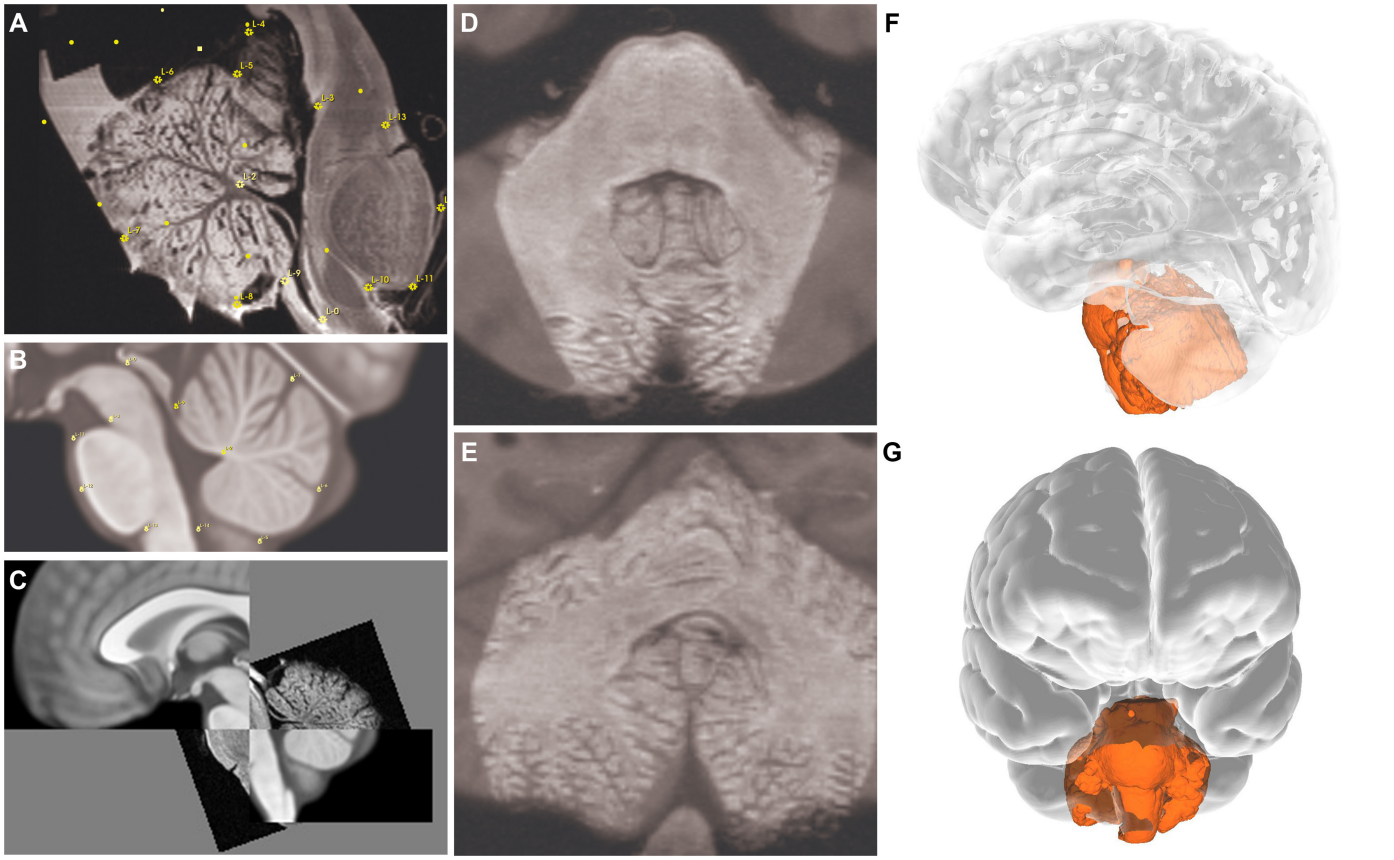
Image planes showing landmarks (*yellow marks*) for the registration process. **(A–C)** Sagittal, **(D)** axial, and **(E)** coronal views. 3D reconstruction of the specimen overlaid on MNI space; **(F)** lateral and **(G)** anterior views. *Used with permission from Barrow Neurological Institute, Phoenix, Arizona*.

The specimen was manually registered to the MNI space using fiducial registration, and accurate anatomical registration was confirmed (Figures 5A, B). The 3D reconstruction provided a precise overlay on the MNI brain model, validating the anatomical accuracy of our findings (Figures 5C–G).

**Figure 6:**
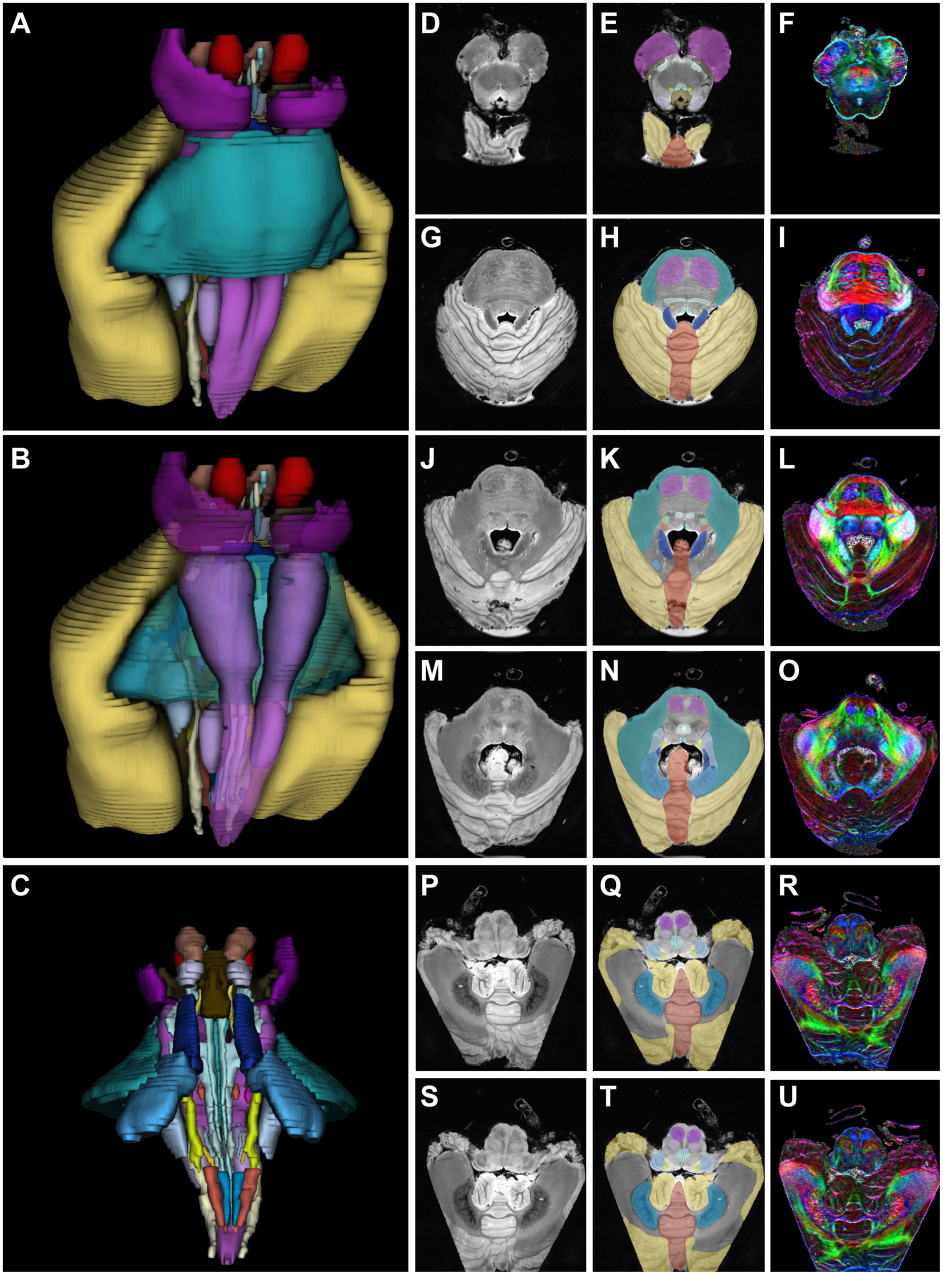
3D reconstruction of segmented structures. Segmentation and color maps of high-resolution fractional anisotropy axial MR images. **(A, D–I)** Mesencephalon. **(B, J–O)** Pons. **(C, P–U)** Medulla. **(D**, **G**, **J**, **M, P, S)** show MR images, **(E**, **H**, **K**, **N**, **Q**, **T)** show segmented structures, and **(F**, **I**, **L**, **O**, **R**, **U)** show fractional anisotropy images of the mesencephalon through the medulla. *Used with permission from Barrow Neurological Institute, Phoenix, Arizona*.

When considering the left and right structures together, 31 different gray and white matter structures were segmented. Their volumes were reconstructed with segmented versions of transverse slices taken from various levels of the medulla, pons, and mesencephalon, along with FA and normal high-resolution images (Figure 6).

#### 3.1.3 White matter tracts and brainstem-cerebellum connectivity

Region-of-interest tracking between the brainstem and cerebellum revealed the SCP, further demonstrating anatomically plausible clusters that conformed to expected anatomical tracts and reinforced the anatomical precision of the tractography.

**Figure 7:**
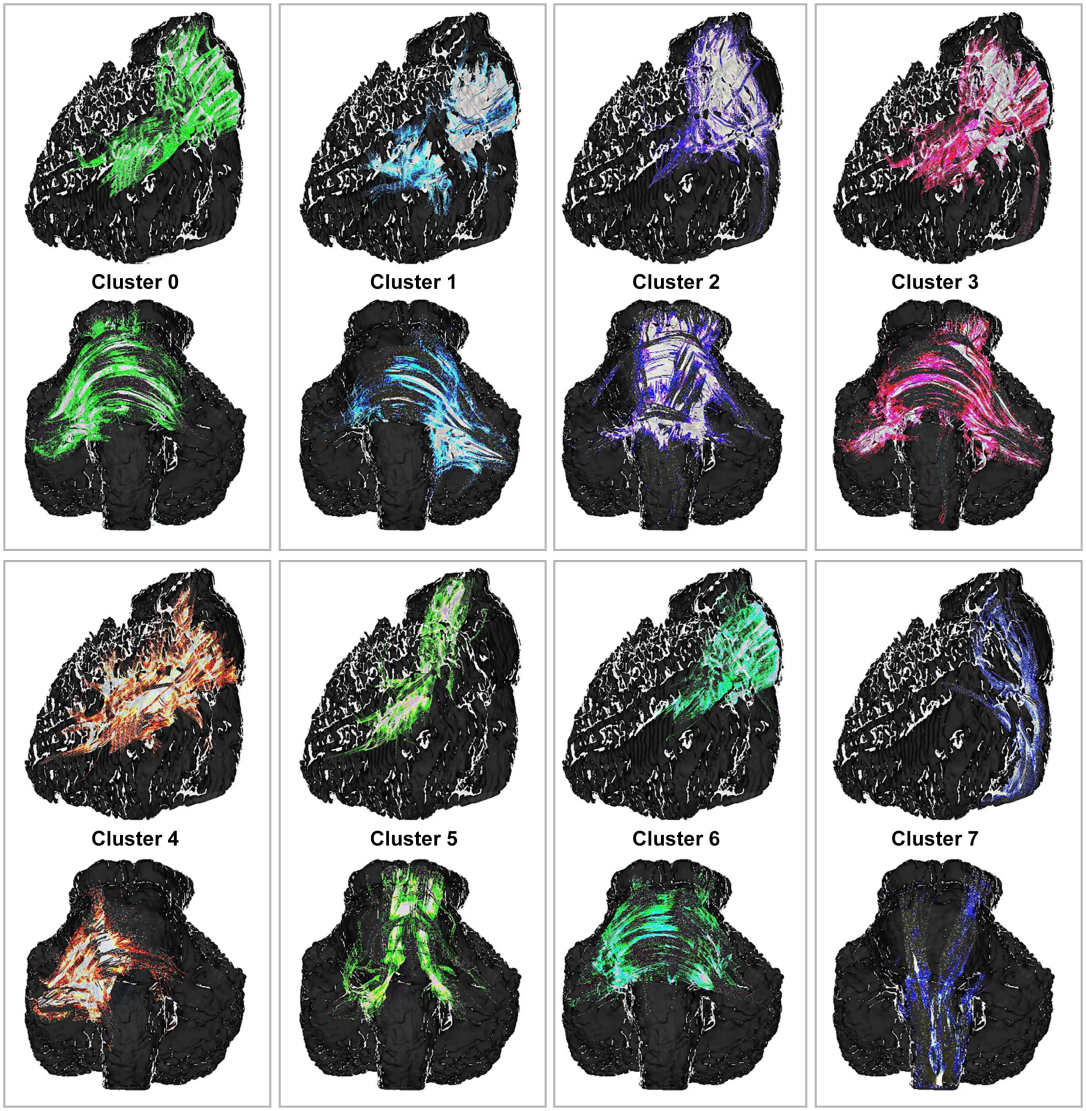
The *k*-means cluster analysis for the region-of-interest tracking. Top and third rows (*lateral view*) and second and fourth rows (*coronal view*) showing anatomically plausible clusters that conformed to expected anatomical tracts and reinforced the anatomical precision of the tractography. The colors were arbitrarily assigned. These clusters are generated based on preferences for left-right and superior-inferior orientations. Although these clusters do not precisely delineate anatomical tracts, they define coherent fiber groups that follow similar trajectories. Anatomical proximities of fibers within the clusters suggest associations with the left middle cerebellar peduncle (cluster 0), right middle cerebellar peduncle (cluster 1), superior cerebellar peduncle (cluster 5), and corticospinal tracts (cluster 7). To the best of our knowledge, the remaining clusters (clusters 2, 3, 4, and 6) demonstrate distinct fiber groups within the middle cerebellar peduncles. *Used with permission from Barrow Neurological Institute, Phoenix, Arizona*.

We used *k*-means clustering to analyze fiber tracts obtained through tractography as a quality control measure, highlighting the physical proximity of these tracts and underscoring their coherence in directional alignment and anatomical course (Figure 7).

Our results revealed the Guillain-Mollaret triangle as a circuit within the brainstem-cerebellum connectivity network. Manual segmentation highlighted the dentate, olivary, and red nuclei of the Guillain-Mollaret triangle in anteroposterior and sagittal views (Figure 4). Furthermore, the 3D reconstruction of the complete tractogram of the Guillain-Mollaret triangle demonstrated the complexity and density of its structures (Supplementary Video 1).

### 3.2 Quantitative results: metrics and measurements

#### 3.2.1 Streamline analysis and mesencephalic-dentate connectivity metrics

A total of 21,641 streamline tracts were generated. The number of streamlines originating from each side, their mean length, and the differences between crossing and noncrossing fibers were analyzed. Mesencephalic-dentate connectivity yielded 5,186 streamlines, with 2,155 originating from the left and 3,031 from the right. The mean length of these streamlines was 69.4 mm; the mean length of crossing fibers was 90.9 mm, and the mean length of noncrossing fibers was 64.1 mm. Consistency was noted in the diameter, tract number, and radius across both right and left crossing and noncrossing fibers (Table 2). Furthermore, the total number of fiber tracts identified in each region was calculated (Table 2) to depict the structural integrity and highlight the differences between the left and right sides of the white matter tracts (Yeh, 2020). This analysis helps us better understand the connectivity within the cerebellar peduncles. Moreover, the mean length and span of the white matter tracts were also calculated, because longer tracts with larger spans (i.e., SCP) play critical roles in long-range communication between different parts of the brainstem and cerebellum (Yeh, 2020).

**Table 2:** Tractographic analysis results of fiber tracts involved in the Guillain-Mollaret triangle.

**Features**	**Inferior cerebellar peduncle left**	**Inferior cerebellar peduncle right**	**Superior cerebellar peduncle left**	**Superior cerebellar peduncle right**	**Central tegmental tract left**	**Central tegmental tract right**
Number of tracts	5,150	5,914	20,887	22,901	2,716	2,070
Mean length (mm)	190.684	166.887	332.394	483.423	231.669	213.152
Span (mm)	411.978	354.228	833.568	890.531	565.217	383.976
Curl	0.46285	0.47113	0.39876	0.542848	0.409876	0.555119
Elongation	325.823	256.796	447.081	814.094	757.718	615.963
Diameter (mm)	585.238	649.884	743.33	593.817	305.746	346.047
Volume (mm^3^)	512.943	553.585	1,443.03	1,338.82	170.09	200.47
Trunk volume (mm^3^)	0	452.194	685.005	446.709	134.315	0.293732
Branch volume (mm^3^)	512.943	508.366	1,374.53	892.113	156.658	200.176
Total surface area (mm^2^)	7,558.45	8,180.49	14,650.8	11,444.1	2,853.1	3,615.53
Total radius of end regions (mm)	113.973	762.597	113.977	116.581	148.238	685.022
Total area of end regions (mm^2^)	158.105	172.974	463.623	482.275	566.162	542.725
Irregularity	215.594	240.088	188.709	126.898	128.215	156.026
Area of end region 1 (mm^2^)	714.844	915.771	295.581	194.58	314.941	287.109
Radius of end region 1 (mm)	627.511	402.349	341.317	828.737	585.514	153.161
Irregularity of end region 1	173.054	555.353	123.819	110.888	341.975	256.684
Area of end region 2 (mm^2^)	866.211	813.965	168.042	287.695	251.221	255.615
Radius of end region 2 (mm)	512.223	360.247	798.456	337.076	896.868	531.861
Irregularity of end region 2	951.578	500.894	119.188	124.071	1,005.89	347.664
Fractional anisotropy	0.19807	0.205582	0.240059	0.264426	0.199156	0.185439
Mean diffusivity (mm^2^/s)	0.2405	0.235121	0.222807	0.215643	0.225164	0.259641
Axial diffusivity (mm^2^/s)	0.290274	0.285696	0.280327	0.278137	0.272418	0.309397
Radial diffusivity (mm^2^/s)	0.215614	0.209834	0.194046	0.184396	0.201538	0.234763

Curl and elongation are shape metrics crucial for visualizing the 3D structure of these tracts (Yeh, 2020). By understanding these values, we can gain insights into any distortions or deformations within the white matter pathways. This is particularly important for evaluating structural changes or asymmetries. The trunk and branch volumes were also calculated to assess potential asymmetries in fiber tracts (Table 2).

FA is a key metric indicating the degree of directionality of water diffusion within the fiber tracts. High FA values indicate high diffusion in one direction, whereas lower values indicate random diffusion in all directions (Stebbins, 2010). To further evaluate the microstructural properties of these tracts, we analyzed diffusivity measures**. **Axial diffusivity represents water diffusion along the fibers, reflecting axonal integrity, whereas radial diffusivity measures diffusion perpendicular to the fibers (Table 2) (Sadeghi et al., 2015).

#### 3.2.2 FA measurements

FA values were calculated, providing quantitative data on the anisotropy of water diffusion within the brainstem tissue (Table 2).

#### 3.2.3 Limitations in quantitative analysis

Some limitations were observed in tractography integrity, such as the nonvisualization of the right-sided cerebellar peduncle decussation, possibly due to inadvertent mesencephalic transection.

## 4 Discussion

In this study, we presented a novel protocol for specimen preparation, scanning, and postprocessing aimed at delineating the intricate anatomy of the human brain and brainstem. Although we used a specimen with a relatively long PMI, our results showed that accurate imaging results can be achieved with proper specimen preparation and MRI techniques, even with longer PMIs. Based on our results, ultrahigh-resolution 7T imaging of *ex vivo* human brain specimens, with proper tissue preservation, imaging techniques, and postimaging processing, is an effective tool for investigating fiber tracts.

Ultrahigh-resolution imaging has emerged as a pivotal development to surmount the limitations inherent in histological and conventional MRI techniques, enhance image quality, and elucidate the complex interactions between white and gray matter (Filley and Fields, 2016). These technical advances are critical to understanding neuroanatomical structures. In particular, the detailed neurosurgical approach to and exploration of brainstem structures, where white and gray matter are intricately and sensitively intertwined and associated with lesions, has gained increasing attention (Guerrero-Gonzalez et al., 2022; Yeh et al., 2021; Hanalioglu et al., 2024; Little et al., 2008; Figueiredo et al., 2016; Cavalcanti et al., 2019, 2020; Garcia-Gonzalez et al., 2012). Our study meticulously analyzed the brainstem and cerebellum to offer unique insights into this evolving field. Using a cadaveric formalin-preserved specimen and a technical design, we highlighted key white and gray matter tracts. This work contributes to a method of convenient understanding and pairing of neurosurgical implications with sensitive brainstem architecture and serves as a foundational model for subsequent surgical anatomical studies to decode the complexities of this essential region (Adil et al., 2021; Agostinelli et al., 2023; Fritz et al., 2019; Lechanoine et al., 2021; Miller et al., 2012; Roebroeck et al., 2019; Rushmore et al., 2020; Shatil et al., 2016; Yendiki et al., 2022).

### 4.1 Implications of extended PMI in diffusion imaging

After death, autolysis degrades tissue quality and can alter certain microstructural characteristics. The PMI between death and chemical fixation appears to be critical for the preservation of tissue quality (Nagy et al., 2015; Tijssen et al., 2022; Sillevis Smitt et al., 1993). Anatomical changes such as myelin loosening can be observed as early as 4 h after death (Krassner et al., 2023; de Wolf et al., 2020), depending on the temperature of the tissue. Radiological signs of autolysis, including a decrease in anisotropy and diffusion, become more pronounced with the lengthening of time between death and fixation, observable within at least 4 h (Thicot et al., 2023). Therefore, rapid fixation is essential for preserving tissue integrity (Fox et al., 1985). Challenges in global laboratory standardization, along with the potentially longer transit times of specimens before postmortem imaging, are crucial factors for obtaining ultrahigh-resolution imaging data from cadavers (Aggarwal et al., 2013; Barrett et al., 2023; Blezer et al., 2007; Oishi et al., 2020; Schilling et al., 2018; Shepherd et al., 2009). We demonstrated that diffusion MRI data and tractography information could be obtained from a cadaver specimen that was formalin-fixed 7 days postmortem and subsequently stored in formalin for 6 months. Our results indicate that proper specimen preparation, MRI techniques, and standardized data collection can yield accurate results even with longer storage times. Furthermore, our use of a higher *b*-value of 3,000 s/mm^2^ increases contrast in both gray and white matter, reflecting the advancements made with the diffusion imaging modalities. Our findings validate earlier studies (Aggarwal et al., 2013; Adil et al., 2021) and show that longer PMIs do not compromise image quality and can still yield sufficient detail in brain tissues to produce reliable images. Thus, our study demonstrates that diffusion imaging can be conducted using human cadavers with extended PMI, making it possible to conduct studies in regions where immediate processing is not feasible.

Our findings also advocate a more nuanced understanding of PMI's effect. Norms between compulsory and fully rigid times on one hand and freedom from any limitation on the other could be reconsidered, as we successfully used a specimen with a 7-day PMI (174 h) stored in formalin without compromising the quality of the diffusion images. Our findings present the possibility of using human *ex vivo* samples that might otherwise have been considered inappropriate to extend the research potential in neuroanatomical studies.

### 4.2 Optimizing brainstem-cerebellum cadaveric specimen preparation

The cadaveric brainstem-cerebellum specimen used in our study underwent formalin fixation at 7 days (174 h) postmortem, followed by long-term formalin storage using standard practices in tissue preservation consistent with the methods outlined by Kim et al. (2021), Massey et al. (2012), Miller et al. (2011), and Tafoya et al. (2017). Our next steps were implemented to accommodate the unique attributes of our sample and our advanced imaging technique. Obtaining cadaveric head-brain specimens with a low PMI is often challenging. In many cases, cadaveric tissues supplied to anatomical laboratories originate from commercial anatomic tissue suppliers and not from intrainstitutional opportunities (i.e., patients who have died in the hospital). Even with in-house anatomic tissue gift or donation programs, the availability of tissues may not be optimal, and such tissues may harbor pathology or disease processes that disrupt the normal anatomy. Thus, acquisition of appropriate tissue often requires special arrangements. The main advance is that cadaveric specimens are often not acquired in an optimal time frame; most cadaveric brain specimens used in neurosurgical anatomical dissection laboratories have been preserved and immersed in formalin solutions, and optimally acquired specimens are infrequently available at best and often associated with considerable cost (>$2,000 each). We have a wealth of experience (>30 years) in neurosurgical anatomical studies (Mignucci-Jiménez et al., 2024; On et al., 2024). We chose a specimen with a relatively long PMI to indicate that, with proper cadaver preservation, custom MRI techniques, and accurate postprocessing, detailed imaging data can be collected even with longer storage times for specimens that will also undergo dissection studies. In addition, at the time, the cadaveric head studied was an intact specimen and in good condition for imaging and correlative anatomic study by dissection. This does not mean that specimens with shorter PMIs would not be usable in the future or for similar studies. For this study, this particular specimen met all the conditions of an optimal head-brain specimen for imaging and dissection.

Specifically, the specimen was rehydrated in a normal saline solution for 7 days prior to imaging. Although not extensively documented, this step is crucial for restoring the tissue's natural hydration, potentially influencing the MRI signal characteristics. Next, on the day of imaging, the specimen was placed in a custom-designed 3D-printed container tailored to fit a 7T MRI pore. This step signifies an advancement over traditional sample holders, such as test tubes or syringe cylinders, because it allows for a more precise fit and minimization of motion artifacts, a concern highlighted in previous studies (Dyrby et al., 2011). Our container featured 2 outlet ports, each connected to a 3-way stopcock filled with Fomblin, one of the inert fluorinated oils commonly used for their MR invisibility and artifact reduction properties (Iglesias et al., 2018). Nonradiosensitive spacers were strategically placed to conserve the amount of inert solution used, an efficiency not commonly reported in existing protocols. Before imaging, we introduced a vacuum degassing process for 30 min, followed by continuous vibration. This approach, which is not explicitly detailed in prior literature, effectively enhanced air bubble removal, a crucial step to avoid image distortion due to magnetic susceptibility differences.

Our protocol leverages an established methodology while introducing innovative steps to optimize specimen preparation for high-resolution MRI (Adil et al., 2021). This approach addresses key challenges such as motion artifacts, air bubble interference, and tissue hydration, thereby enhancing the quality and reliability of the imaging data. Our work thus demonstrates the potential of customized specimen preparation methods in advancing neuroimaging research.

### 4.3 MRI protocol for brainstem and cerebellum

Our novel MRI protocol for cadaveric brain specimens draws upon foundational principles for DTI and diffusion kurtosis imaging yet introduces specific modifications to enhance imaging precision.

Our extended anatomic imaging cycle, with a duration of 3 h and 28 min and a high resolution of 78.125 × 78.125 × 500 mm3, is designed to capture the intricate details of the brainstem and cerebellum. This approach aligns with Dyrby et al. (2011), who emphasize the importance of detailed imaging for accurate fiber reconstruction. The extended duration and high resolution align with the recommendations of Henriques et al. (2020) for capturing subtle anatomical variation. The DTI protocol, lasting 48 h and 48 min with a *b*-value of 3,000 s/mm^2^ across 60 directions, significantly exceeds the typical *b*-value ranges suggested for *ex vivo* imaging. This higher *b*-value, as supported by Maffei et al. (2022) and Jones et al. (2020) is crucial for enhancing the contrast and angular resolution to enable more precise mapping of complex fiber architectures.

We aimed to strike a balance between high-resolution imaging and scan duration, and with the sequences used, we achieved these ultrahigh-resolution anatomical imaging results in a relatively convenient 48 h and 48 min. We chose our TR/TE to limit the overall stress put on our gradient equipment. Reducing the number of targeted slices would shorten the scan time, but the duration also depends on the sample; scanning a larger sample would require longer scan times. Increasing the resolution or diffusion directions will also lengthen scan time. In other studies, we are doping the specimen in a solution with gadolinium, which causes an increase in the scan time; however, our results are still under analysis. In summary, scan time depends on many factors. Other studies may achieve high-resolution imaging with longer scan durations. Although this protocol has already provided us with high-resolution imaging, longer scan durations using gadolinium for contrast enhancement may be required in future studies.

We used a deterministic algorithm with an advanced technique for identifying and tracking neural pathways in DSI Studio for tractography. The specific parameters set for fiber tracking, including angular thresholds and track lengths, ensured accuracy and relevance in the pathways identified. This approach to tractography, influenced by the work of Schilling et al. (2018), Grisot et al. (2021), and Yendiki et al. (2022), demonstrates our ability to provide detailed and reliable interpretations of neural structures using our protocol.

### 4.4 Differences from existing protocols

Ultrahigh-resolution anatomic MRI and DTI of formalin-fixed *ex vivo* human brain have been previously studied by Paxinos et al. (2023) and Naidich et al. (2009). They successfully obtained detailed anatomical images using different protocols (Table 3). However, they encountered challenges such as longer scan times and artifacts. To address these challenges, we captured ultrahigh-resolution anatomical images while optimizing scan times and minimizing artifacts (Table 3). Our protocol differs from those protocols in several important perspectives (Paxinos et al., 2023; Naidich et al., 2009). First, in terms of specimen preparation, the specimen in our study had a PMI of 174 h, followed by a 6-month formalin fixation. The specimen was then rehydrated in normal saline for 7 days before imaging, which may help wash out free formalin to optimize tissue quality for imaging. Instead of using saline perfusion before formalin fixation, as is used in other protocols (Paxinos et al., 2023), we opted for passive rehydration with normal saline after fixation, 1 week before imaging. Additionally, we used a 4% formalin solution rather than the 10% and 15% solutions used in other protocols (Paxinos et al., 2023; Naidich et al., 2009). We have also performed a degassing protocol to decrease the artifacts (Figure 1).

**Table 3:** Comparison of protocols used by 3 research groups for ultrahigh-resolution imaging of formalin-fixed *ex vivo* human brain specimens.

**Feature**	**Paxinos et al. (2023)**	**Naidich et al. (2009)**	**Current study**
Specimen preparation	Perfused with saline *ex vivo*, immersion fixed in 10% formalin, soaked in neural phosphate buffered saline doped with 2.5 mM gadoteridol for 3 weeks	10 formalin-fixed cadaver tissue samples fixed in 15% formalin for 2 weeks, suspended in phosphate buffer, and then imaged	PMI 168 h, 4% formalin fixation for 6 months, followed by rehydration with normal saline for 7 days prior to imaging
Field strength	7T	9.4T	7T
Magnet type	Agilent Direct Drive MRI system (Agilent Technologies, Santa Clara, CA)	Bruker Magnex superconducting magnet with 89-mm vertical bore (Bruker Analytik, Rheinstetten, Germany)	Bruker Biospec 70/30, with 30-cm bore size (Bruker Corporation, Billerica, MA)
RF coil	65 × 100-mm quadrature RF coil	30-mm bird cage coil	70-mm volume coil
Pulse sequence	Simple diffusion-weighted spin echo pulse sequence TR/TE = 100 ms/33.6 ms, BW = 278 Hz/pixel	Pulsed gradient spin echo sequence: TR = 12,000 ms, TE = 25.7 ms	Custom sequence for DTI and FLASH for anatomical imaging, TR/TE = 3,500/15 ms for sagittal, 1,500/9 ms for coronal and axial
*b*-value	4,000 s/mm^2^	1,600 s/mm^2^	3,105 s/mm^2^
Gradient settings	δ = 4.7 ms, Δ = 26 ms	Not specified	δ = 7 ms, Δ = 14 ms
Diffusion directions	120 directions	6 directions	60 directions
Voxel size	200 μm isotropic	1-mm slice thickness, data matrix: 128 × 128	In-plane resolution 0.55 mm, slice thickness 0.5 mm
Scan duration	208 h	24 h for 24 slices	48 h and 48 min
Data analysis software	Custom postprocessing pipelines	DtiStudio v2.4	DSI Studio, DIPY
Tractography method	Not specified	FACT algorithm	Deterministic algorithm in DSI Studio
Additional imaging	High-resolution GRE images	Color maps and eigenvector maps for visualization	High-resolution anatomical imaging with FLASH sequence

Δ, separation; δ, width; BW, bandwidth; DIPY, diffusion imaging in phyton documentation; DSI, diffusion spectrum imaging; DTI, diffusion tensor imaging; FACT, fiber assignment by continuous tracking; FLASH, fast low-angle shot; GRE, gradient echo; MRI, magnetic resonance imaging; PMI, postmortem interval; TE, time to echo; TR, repetition time.

Similar to other MRI study acquisitions, we used a 7T preclinical scanner (Paxinos et al., 2023). Although higher field strengths can offer better signal-to-noise ratios (Naidich et al., 2009), differences in diffusion directions can offset these advantages. After DTI, we used the fast low-angle shot sequence for anatomical imaging to reduce the sequence interval instead of other sequences used in other protocols (Adil et al., 2021; Naidich et al., 2009). This combination enabled us to achieve high-resolution anatomical and DTI data, optimizing the visualization of fine neuroanatomical structures with a shorter scan time.

Our protocol included 60 diffusion directions, providing a robust DTI and tractography dataset and allowing for accurate mapping of white matter tracts. As seen in other protocols, increasing the number of diffusion directions produces more detailed tractography results but causes extremely long scan times (Paxinos et al., 2023). Regarding voxel size, our protocol achieved an in-plane resolution of 0.55 mm with a slice thickness of 0.5 mm, offering intricate detail for DTI and anatomical imaging. Using finer voxel sizes causes extended scan times (Paxinos et al., 2023). Thus, we selected a voxel size that balances detail with practical scanning duration.

We aimed to strike a balance between high resolution and reasonable scan times. Our protocol offers advanced diffusion imaging and tractography with 60 diffusion directions, providing detailed anatomical imaging without the impractical scan times associated with other protocols. As such, our protocol may be an optimal choice for detailed neuroanatomical studies, particularly for presurgical trajectory planning studies, considering both time efficiency and resolution. Furthermore, although we used a specimen with a relatively long PMI in our study, we still obtained ultrahigh-resolution imaging results and intricately visualized the crucial anatomical structures and white matter tracts of the brainstem-cerebellum complex. However, further studies with more samples, particularly with short PMIs, are needed to refine and optimize the protocol.

### 4.5 Anatomic segmentation

Although we performed manual segmentation in our study, other options exist, such as automatic image segmentation with the aid of artificial intelligence (Mofatteh, 2021; Khalili et al., 2019). Even though consulting the neuroanatomy atlases significantly improves precision, automatic segmentation using artificial intelligence algorithms may decrease the risk of subjectivity and potential errors. On the other hand, manual segmentation depends on the interpreter's neuroanatomical knowledge and may be time-consuming. Automatic segmentation can address this issue with a success rate similar to that of physicians (Mofatteh, 2021; Dolz et al., 2016; Yamashita et al., 2008). The automatic segmentation methods are mainly preferred in the preoperative planning of high-risk brainstem tumor surgery and in localizing the epileptogenic zone in epilepsy surgery (Mofatteh, 2021; Kassahun et al., 2014).

### 4.6 Limitations

In this study, a single specimen was used, which limits the generalizability of our findings. Future studies with a larger number of specimens are necessary to enhance the robustness of the results. Although we could obtain detailed neuroanatomical images using a specimen with a relatively long PMI, it will be essential to include specimens with shorter PMIs in future studies to assess potential differences in tissue quality and imaging outcomes. Moreover, studies that match PMI with tissue quality and MRI data are needed to establish the optimum PMI without compromising tissue morphology.

## 5 Conclusions

We developed and validated a specific protocol that enabled successful ultrahigh-resolution MRI and DTI of an *ex vivo* human brainstem-cerebellum with a 1-week PMI and long-term formalin preservation. The protocol provided a detailed visualization of the anatomic structures of the brainstem-cerebellum sections at a microscopic level, as well as of white matter tracts and high-precision segmentation of crucial brainstem and cerebellum structures, delineating the mesencephalic-dentate connectivity and the Guillain-Mollaret triangle. High-resolution imaging of the brainstem is of high importance for understanding the anatomical implications of various neurosurgical approaches to the brainstem and cerebellum.

## Data Availability

The original contributions presented in the study are included in the article/Supplementary material, further inquiries can be directed to the corresponding author.

## Supplementary material

Supplementary Table 1 Color codes of segmented anatomical structures

Supplementary Video 1 The 3-dimensional dynamic images of a full tractogram of the specimen indicating the complexity and density of its structures and tracts. The dynamic image on the right side demonstrates the nuclei and the fiber tracts involved in the Guillain-Mollaret triangle. *Used with permission from Barrow Neurological Institute, Phoenix, Arizona*.
