# Supplementary material for: Ultrahigh-resolution 7-Tesla anatomic magnetic resonance imaging and diffusion tensor imaging of ex vivo formalin-fixed human brainstem-cerebellum complex
Source: Front Hum Neurosci. 2024 Nov 27;18:1484431. doi: 10.3389/fnhum.2024.1484431 (PMC11631901; doi:10.3389/fnhum.2024.1484431)
Supplement: Supplementary Table 1 — Color codes of segmented anatomical structures [file Table_1.docx]

**Supplemental Table 1.** Color codes of segmented anatomical structures

| **Color code** | **Region Name** | **Brainstem region** | **Color** |
| --- | --- | --- | --- |
| #e40909 | Red_nucleus | Mesencephalon |  |
| #3b3423 | Substantia_nigra | Mesencephalon |  |
| #b17b63 | Superior_colliculus | Mesencephalon |  |
| #c8c8ea | Inferior_colliculus | Mesencephalon |  |
| #e4de8f | Trochlear_nucleus | Mesencephalon |  |
| #fffadc | Oculomotor_nucleus | Mesencephalon |  |
| #e4dd46 | Mesencephalic_nucleus_of_trigeminal | Mesencephalon |  |
| #900eb0 | Cerebral_crus | Mesencephalon |  |
| #483723 | Periaqueductal_gray | Mesencephalon |  |
| #d8654f | Abducens_nucleus | Pons |  |
| #d31ac4 | Facial_nucleus | Pons |  |
| #90ee90 | Motor_nucleus_of_trigeminal | Pons |  |
| #c06858 | Principal_nucleus_of_trigeminal | Pons |  |
| #dcf514 | Vestibular_complex | Pons and medulla |  |
| #fafad2 | Spinal_nucleus_of_trigeminal | Medulla |  |
| #faca1c | Nucleus_tractus_solitarius | Medulla |  |
| #0097d1 | Nucleus_gracilis | Medulla |  |
| #d66553 | Nucleus_cuneatus | Medulla |  |
| #b79ddc | Inferior_olivary_complex | Medulla |  |
| #b6d6d3 | Hypoglossal_nucleus | Medulla |  |
| #95bfcd | Dorsal_vagal_nucleus | Medulla |  |
| #6cbad0 | Accessory_nucleus | Medulla |  |
| #b2d3f6 | Inferior_cerebellar_peduncle | Medulla |  |
| #26a5ac | Middle_cerebellar_peduncle | Pons |  |
| #1b43c6 | Superior_cerebellar_peduncle | Mesencephalon |  |
| #e4cd79 | Cerebellar_cortex | Cerebellum |  |
| #d85237 | Vermis | Cerebellum |  |
| #55bcff | Dentate_nucleus | Cerebellum |  |
| #b7ebf5 | Medial_leminiscus | White matter |  |
| #adf9f7 | Medial_longitudinal_fasciculus | White matter |  |
| #b05bc7 | Corticospinal_tract | White matter |  |
